# Swept source optical coherence tomography to early detect multiple sclerosis disease. The use of machine learning techniques

**DOI:** 10.1371/journal.pone.0216410

**Published:** 2019-05-06

**Authors:** Amaya Pérez del Palomar, José Cegoñino, Alberto Montolío, Elvira Orduna, Elisa Vilades, Berta Sebastián, Luis E. Pablo, Elena Garcia-Martin

**Affiliations:** 1 Group of Biomaterials, Aragon Institute of Engineering Research (I3A), University of Zaragoza, Zaragoza, Spain; 2 Department of Mechanical Engineering, University of Zaragoza, Zaragoza, Spain; 3 Department of Ophthalmology, Miguel Servet University Hospital, Zaragoza, Spain; 4 GIMSO Research and Innovative Group, Aragon Institute for Health Research (IIS Aragón), University of Zaragoza, Zaragoza, Spain; 5 Department of Neurology, Miguel Servet University Hospital, Zaragoza, Spain; Bascom Palmer Eye Institute, UNITED STATES

## Abstract

**Objective:**

To compare axonal loss in ganglion cells detected with swept-source optical coherence tomography (SS-OCT) in eyes of patients with multiple sclerosis (MS) versus healthy controls using different machine learning techniques. To analyze the capability of machine learning techniques to improve the detection of retinal nerve fiber layer (RNFL) and the complex Ganglion Cell Layer–Inner plexiform layer (GCL+) damage in patients with multiple sclerosis and to use the SS-OCT as a biomarker to early predict this disease.

**Methods:**

Patients with relapsing-remitting MS (n = 80) and age-matched healthy controls (n = 180) were enrolled. Different protocols from the DRI SS-OCT Triton system were used to obtain the RNFL and GCL+ thicknesses in both eyes. Macular and peripapilar areas were analyzed to detect the zones with higher thickness decrease. The performance of different machine learning techniques (decision trees, multilayer perceptron and support vector machine) for identifying RNFL and GCL+ thickness loss in patients with MS were evaluated. Receiver-operating characteristic (ROC) curves were used to display the ability of the different tests to discriminate between MS and healthy eyes in our population.

**Results:**

Machine learning techniques provided an excellent tool to predict MS disease using SS-OCT data. In particular, the decision trees obtained the best prediction (97.24%) using RNFL data in macular area and the area under the ROC curve was 0.995, while the wide protocol which covers an extended area between macula and papilla gave an accuracy of 95.3% with a ROC of 0.998. Moreover, it was obtained that the most significant area of the RNFL to predict MS is the macula just surrounding the fovea. On the other hand, in our study, GCL+ did not contribute to predict MS and the different machine learning techniques performed worse in this layer than in RNFL.

**Conclusions:**

Measurements of RNFL thickness obtained with SS-OCT have an excellent ability to differentiate between healthy controls and patients with MS. Thus, the use of machine learning techniques based on these measures can be a reliable tool to help in MS diagnosis.

## Introduction

Multiple sclerosis (MS) is a neurodegenerative disease of the central nervous system that disrupts the flow of information within the brain, and between the brain and body. The cause of MS is still unknown and the progress, severity and specific symptoms of MS in any one person cannot yet be predicted. Most people with MS are diagnosed between the ages of 20 and 50, with at least two to three times more women than men being diagnosed with the disease. However, an early diagnosis is determinant to slow down the progression of the disease [1]. MS is diagnosed on the basis of clinical findings and supporting evidence, such as magnetic resonance imaging (MRI) of the brain and spinal cord and cerebrospinal fluid examination [2], but these tests are invasive and expensive, so they are performed when there are evidences of the disease but not in a routine way. However, in the last years, different studies suggest that retinal optical coherence tomography (OCT) should be used as a complement to MRI for the analysis of neurodegeneration process in MS [3][4].

Following this line, many studies [5] have reported a correlation between axonal loss in the optic nerve of the retina and MS. The retina can be considered as an extension of the central nervous system (CNS) [6]; it consists of retinal ganglion cells (RGCs) and their axons, known as retinal nerve fiber layer (RNFL), which are in fact CNS axons form the optic nerve. Thus, various eye-specific pathologies share characteristics of other CNS pathologies. Inflammatory disorders, such as multiple sclerosis (MS), and degenerative diseases, such as Alzheimer’s disease (AD) and Parkinson’s disease (PD), also show pronounced axonal pathology. In fact, it is recognized that significant RGCs and RNFL loss occurs in patients with CNS pathologies. It has been demonstrated that in patients suffering from CNS diseases, the thickness of the RNFL significantly decreases as the disease goes on [7].

Loss of retinal ganglion cells can be detected using ocular imaging technologies such as optical coherence tomography (OCT). Petzold et al. [8] showed that neuroaxonal injury can be detected with OCT measuring RNFL and GCL thickness decrements relative to normal control subjects. OCT provides a noninvasive, rapid, objective and reproducible method for evaluating the different layers of the retina [7]. Specifically, the layers that are part of the central nervous system are the retinal ganglion cell (GCL+) and the retinal nerve fiber layer (RNFL). OCT allows for cross-sectional imaging of the retina and the optic disc based on interference patterns produced by low coherence light reflected from retinal tissues [9][10]. The first description of reduced peripapilary retinal nerve fiber layer thickness in MS patients was made by Parisi et al. [11] using older third generation time domain OCT. Since then, the evolution of these platforms has provided accurate segmentation processes to quantify discrete retinal layers [4]. Actually, the technique really improved when Fourier-domain versions of OCT were introduced. Two main variations of Fourier-domain OCT have been developed over the last years: spectral-domain OCT (SD-OCT) and swept-source OCT (SS-OCT). The difference between them comes from the mechanism employed to measure interference corresponding to different frequencies. There are several different SD–OCT machines commercially available, such as RTVue (Optovue, Fremont, CA, USA), Spectralis OCT (Heidelberg Engineering, Dossenheim, Germany), and Cirrus SD–OCT (Carl Zeiss Meditec, Dublin, CA, USA). Here, SS-deep range imaging (DRI) OCT Triton (Topcon, Tokyo, Japan) was used and the main protocols of this device were analyzed to evaluate their ability to diagnose MS.

OCT studies performed in MS patients have proved a significant thinning of both RNFL and ganglion cell–inner plexiform layer (GCL+) [4]. However, some authors [12] have demonstrated that GCL+ thickness measures have better reliability and reproducibility than RNFL thickness measures. More recently, Martinez-Lapiscina et al. [5] studied a large cohort of 879 patients with clinically isolated syndrome (CIS) of MS with baseline OCT and clinical follow-up. This study found that baseline RNFL thickness give a measure of the risk of disability progression.

In this paper we explore the use of machine learning techniques to detect the disease using data from SS-OCT. To the best of our knowledge, this is the first study evaluating MS eyes using swept source OCT technology in combination with machine learning techniques. Some preliminary studies [13][14] have analyzed the role of these techniques using specific protocols and other OCT devices. Here, different protocols obtained by DRI OCT Triton were analyzed and compared in order to specify the success ratio of different machine learning techniques for determining MS disease.

The final goal of this research is to obtain a simple classification algorithm that could be implemented in the OCT device software using only data from baseline visit. Thus, the OCT technique could be an easy, non-invasive and costless test that could be used in routine explorations in order to help neurologists in early detect MS disease.

## Material and methods

### Subjects and measurement protocol

The experimental protocol was approved by the Ethics Committee of the Miguel Servet Hospital, and all participants provided written informed consent to participate in this research. This study adhered to the tenets of the Declaration of Helsinki.

Two independent samples of 180 healthy controls (114 female and 66 male) and 80 patients (54 female and 26 male) with relapsing-remitting (RR) MS were prospectively recruited in this observational cross-sectional study. The diagnosis of MS was based on standard clinical and neuroimaging criteria: objective demonstration of dissemination of lesions in both time and space and magnetic resonance imaging is integrated with clinical and other paraclinical diagnostic methods [15]. Related medical records were carefully reviewed for information regarding disease duration, the Expanded Disability Status Scale (EDSS), disease-modifying treatments, acute MS attacks, and the presence of prior episodes of optic neuritis as reported by the treating neurologist and patient. The diagnosis of prior episodes of optic neuritis (ON) was based on clinical findings, which included the presence of decreased visual acuity, a visual field defect, color vision loss, relative afferent pupil defect and a compatible fundus examination [16].

Required inclusion criteria were as follows: best-corrected visual acuity (BCVA) of 20/40 or better, refractive error within ±5.00 diopters equivalent sphere and ±3.00 diopters astigmatism, transparent ocular media (nuclear color/opalescence, cortical or posterior subcapsular lens opacity <1) according to the Lens Opacities Classification System III system [17]. Exclusion criteria included previous intraocular surgery, diabetes or other diseases affecting the visual field or neurological system, and current use of medications that could affect visual function. All participants underwent a full ophthalmologic examination: clinical history, visual acuity, biomicroscopy of the anterior segment using a slit lamp, Goldmann applanation tonometry and ophthalmoscopy of the posterior segment. One eye from each patient/healthy control was randomly selected (excluding eyes with previous episodes of ON) [18].

A total of 258 eyes of white European origin (179 from healthy individuals and 79 from patients with MS) were included in the analysis.

**Fig 1:**
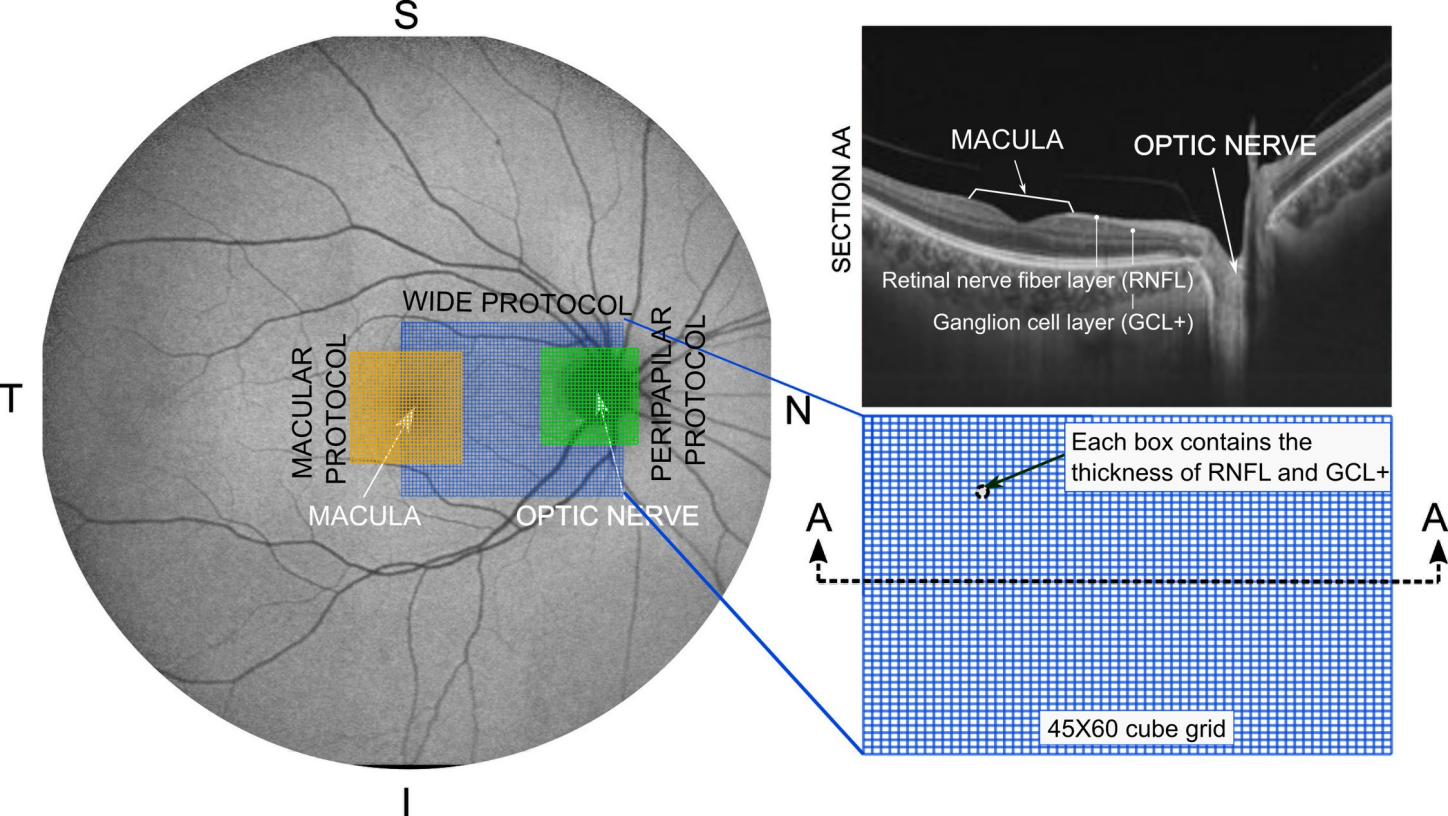
Schematic representation of the different analyzed protocols. The used protocols are shown on a retina image of a right eye (N: nasal; T: temporal; S: superior; I: inferior). The grid of the macular protocol is composed of 30x30 cubes centered in the fovea. The peripapilar protocol is centered in the optic nerve and it is composed of a 26x26 cube grid. A detail of the wide protocol which covers an area between macula and optic nerve using a 45x60 cube-grid is shown. The OCT software makes a scan of this area determining the thickness of the different retinal layers. In section AA, a cross section of the retina, where the retinal nerve fiber layer (RNFL) and the complex ganglion cell layer–inner plexiform layer (GCL+) are highlighted, is shown. For all cases, each box measures 200x200μm. As shown, each box contains the thickness of the different layers of the retina but in this work only data from RNFL and GCL+ were used.

Structural measurements of the retina were acquired using the DRI Triton SS-OCT device (Topcon, Tokyo, Japan), which is a multi-modal swept source OCT with a non-mydriatic color fundus camera. This device utilizes a 1,050 nm wavelength and reaches a scanning speed of 100,000 A-scans per second, with 8 and 20 μm axial and transverse resolution in tissue, respectively. The scan provides separate thickness measurements of different retinal layers: retinal nerve fiber layer (RNFL) (between the inner limiting membrane (ILM) to the ganglion cell layer boundaries), ganglion cell layer (GCL) + (between RNFL to the inner nuclear layer boundaries), GCL++ (between ILM to the inner nuclear layer boundaries), and retinal thickness (from the ILM to the retinal pigment epithelium boundaries). Here, OCT scans were performed to obtain measurements of the macular, peripapilar and macular-peripapilar RNFL and GCL+ thicknesses (see Fig 1) using three of the main protocols of DRI OCT Triton device: Wide protocol 3D(H) + 5 LineCross 12x9mm Overlap 8; Macular protocol 3D Macula(H) 7x7mm; and Peripapilar protocol 3D Disc 6x6mm. All scans were performed by the same experienced operator, who was blinded for presence or not of MS in each subject.

## Data analysis

All variables were registered in a database created with Excel 2010 (Microsoft Corporation). Statistical analysis was performed using commercial predictive analytics software (SPSS, version 20.0; SPSS, Inc., Chicago, IL). The normality of the samples distribution (MS patients and healthy controls) was confirmed using the Kolmogorov–Smirnov test (p>0.05). Comparison between data from healthy controls and MS patients was performed for each age group and for each layer using a t-Student analysis. The mean and standard deviation values of RNFL and GCL+ thicknesses were compared between subjects using wide, macular and peripapilar protocols. To obtain the p-value in each test, the Levene test statistic was previously computed. A significant difference between groups was considered for p<0.05.

### Machine learning algorithms

We evaluated the performance of the machine learning algorithms for classifying RNFL and GCL+ thickness measurements obtained with the DRI OCT Triton as MS or healthy. Machine learning algorithms are data mining tools used fundamentally to look for patterns in training sets of data; these algorithms learn these patterns and develop the ability to accurately classify new patterns. In this study, WEKA [19] software was used and several supervised learning models were used. The same inputs and outputs were introduced in all cases. As inputs, it was considered that there is one class for sex (female-male), one attribute for the patient age and so many attributes as grid boxes (each box contains RNFL or GCL+ thickness) depending on the analyzed protocol (see Fig 1). Every attribute was normalized before entering in the data mining tool. As output, only one class for classifying the disease (YES-MS, NO-HEALTHY) was used.

First, a feed-forward neural network trained by a back-propagation algorithm multilayer perceptron (MLP) was tested [20]. The structure of MLP is based on different layers of nodes or neurons. There is always an input layer with so many neurons as inputs of the model and an output layer with the same number of neurons as outputs. Furthermore, the hidden layer can be unique or several hidden layers can be defined. The structure of the MLP cannot be predefined and it highly depends on the problem to be solved. Here, the architecture of the MLP was different for each protocol, but the same rule was used to construct the hidden layer (only one hidden layer was considered). The number of neurons in the hidden layer was calculated using the following rule, number of neurons = (attributes + classes)/2.

The support vector machine (SVM) tools were also analyzed [21]. These tools are supervised learning models that analyze data used for classification and regression analysis. An SVM model is a representation of the different data as points in space, mapped so that the data of the separate categories (MS or healthy) are divided by a clear gap that is as wide as possible. Given a set of training examples, each marked as belonging to MS or healthy category, this machine learning algorithm develops a model that assigns new data to one category or the other. SVMs can efficiently perform a non-linear classification using what is called the kernel trick, implicitly mapping their inputs into high-dimensional feature spaces. In this research, different kernel functions were used: linear, polynomial, radial basis and sigmoid.

The ability of decision trees to accurately classify between healthy controls (HCs) and MS patients was investigated too. A decision tree is a flowchart-like structure in which each internal node represents a "test" on an attribute, each branch represents the outcome of the test, and each leaf node represents a class label (decision taken after computing all attributes). The paths from root to leaf represent classification rules. Here, C4.5 decision tree [22] and random forest [23] among others, were analyzed.

The above mentioned algorithms were also tested including an Attribute Classifier algorithm before. In order to find the importance of the attributes (or the inputs), feature selections algorithms are used. Thus, instead of processing all the attributes only relevant attributes are involved in the mining process. This kind of algorithms are designed to reduce the dimensionality, remove irrelevant and redundant data. Thus, in this case, only the most significant attributes fed the machine learning algorithm.

Finally, and taking into account that the data is very disperse among patients, the Adaptive Boosting (machine learning meta-algorithm) formulated by Freund and Schapire [24] was used in conjunction with the above mentioned learning algorithms to improve performance. Boosting is a general ensemble method that creates a strong classifier from a number of weak classifiers. In particular, AdaBoost is sensitive to noisy data and outliers. In some problems it can be less susceptible to the overfitting problem than other learning algorithms.

To compare among algorithms and combinations, a 10-fold cross validation resampling method was used. Cross validation is a technique to evaluate predictive models by partitioning the original sample into a training set to train de model and a test set to evaluate it. Here, all data were randomly divided into 10 subsets. Of the 10 subsets, a single subset is retained as the validation data for testing the model and the remaining 9 subsets are used as training data. The cross-validation is then repeated 10 times (the folds), with each of the 10 subsets used exactly once as the validation data. The 10 results from the folds are averaged to produce a single estimation. The prediction rate was analyzed as well as the receiver operating characteristic curve, i.e. ROC curve, a graph of a function that illustrates the diagnostic ability of a binary classifier system. The ROC curve is created by plotting the true positive rate (TPR) against the false positive rate (FPR) at various threshold settings. The true-positive rate is also known as sensitivity or probability of detection in machine learning.

## Results

### Subjects and measurement protocol

MS Seventy-nine eyes from relapsing-remitting (RR) MS patients with a mean age of 45.53 years (SD = 13), and 179 eyes from healthy individuals with a mean age of 49.02 years (SD = 15.34) were included in the study. Male/female ratio was 0.48 in the MS group and 0.57 in the control group. The intraocular pressure was 14.51±3.02 mm Hg in MS group and 14.77±2.87 mm Hg in healthy controls. Age, sex and intraocular pressure did not differ significantly between the groups (p = 0.222, 0.213, 0.520 respectively). Disease duration in the group of patients was 7.12 years (SD = 2.66). The median EDSS score was 2.52 (IQR = 0.53) and all patients suffered from relapsing-remitting MS subtype. All individuals in both cohorts were Caucasians.

### Data analysis

**Fig 2:**
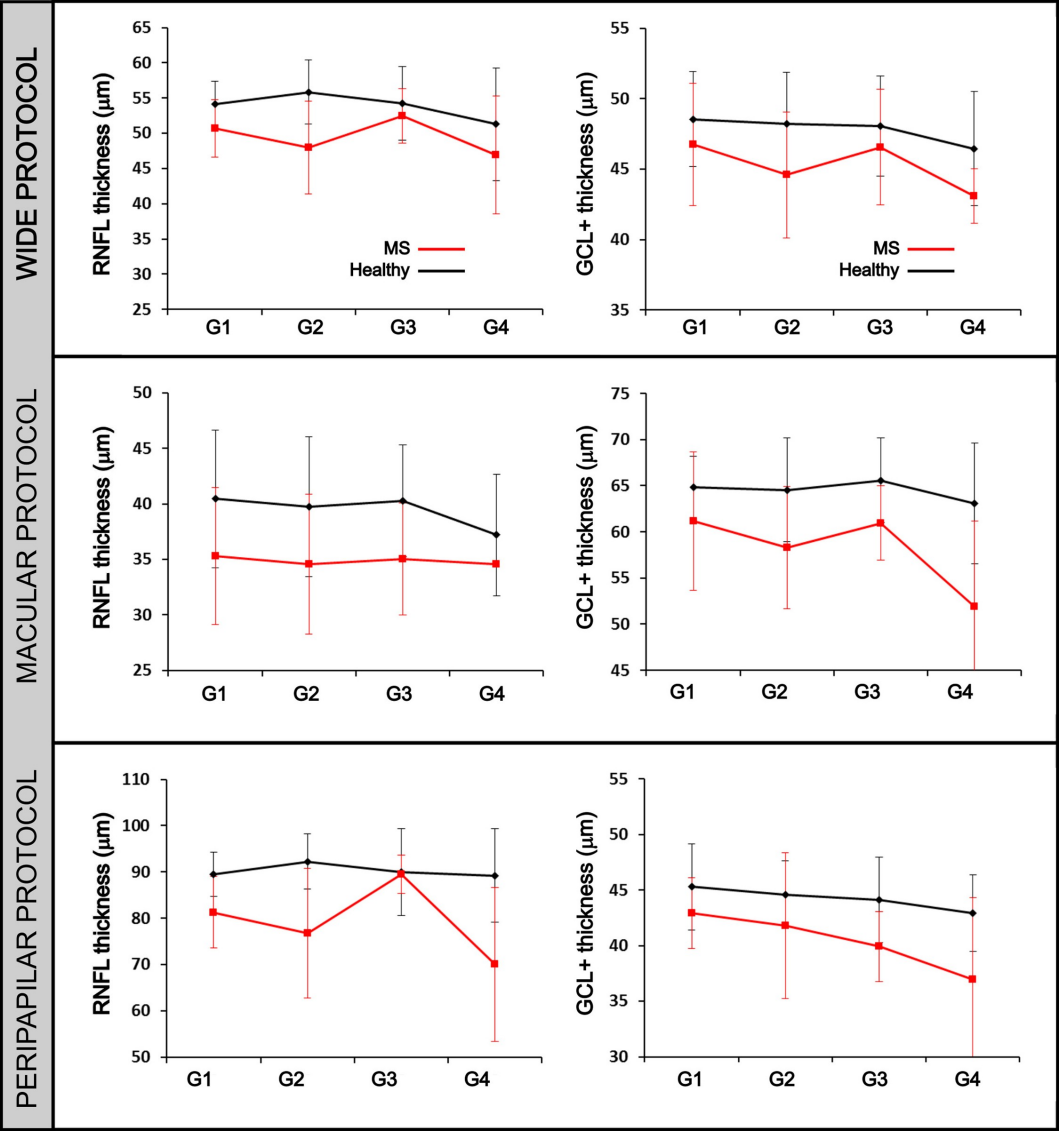
RNFL and GCL+ thickness data. Average retinal nerve fiber layer (RNFL) and complex ganglion cell layer–inner plexiform layer (GCL+) thicknesses for age group measured by different protocol.

The average thickness of the RNFL and GCL+ were computed for different age groups and for different protocols (see Fig 2 and Table 1). For an extended graphical information of the statistical analysis, refer to the Supplementary Material (S1 Fig).

**Table 1:** Statistical analysis of the differences between healthy controls and MS patients in retinal layers thicknesses. P-value obtained by t-student test comparing the average thickness of RNFL and GCL+ of healthy controls (HC) and multiple sclerosis patients (MS) for each age group and different protocol. Mean and standard deviation thickness values shown in μm.

**WIDE**	**RNFL LAYER**							
**PROTOCOL**	G1		G2		G3		G4	
	HC	54.28±3.15	HC	55.61±4.46	HC	54.25±5.22	HC	51.19±8.10
	MS	50.70±4.05	MS	47.96±6.56	MS	52.44±3.88	MS	46.90±8.34
	p = 0.007		p = 0.0		p = 0.218		p = 0.194	
	**GCL+ LAYER**							
	G1		G2		G3		G4	
	HC	48.56±3.40	HC	48.06±3.67	HC	48.03±3.58	HC	46.39±4.10
	MS	46.75±4.34	MS	44.58±4.19	MS	46.55±3.99	MS	43.01±2.09
	p = 0.187		p = 0.001		p = 0.129		p = 0.059	
**MACULAR**	**RNFL LAYER**							
**PROTOCOL**	G1		G2		G3		G4	
	HC	40.59±3.95	HC	39.59±3.93	HC	40.31±4.32	HC	37.01±3.79
	MS	35.29±6.18	MS	34.57±6.30	MS	35.04±5.04	MS	34.60±5.46
	p = 0.035		p = 0.0		p = 0.005		p = 0.165	
	**GCL+ LAYER**							
	G1		G2		G3		G4	
	HC	64.85±3.39	HC	64.37±5.63	HC	65.60±4.60	HC	62.97±6.64
	MS	61.14±7.50	MS	58.30±6.61	MS	60.94±4.02	MS	51.88±9.29
	p = 0.182		p = 0.0		p = 0.003		p = 0.001	
**PERIPAPILAR**	**RNFL LAYER**							
**PROTOCOL**	G1		G2		G3		G4	
	HC	89.74±4.54	HC	91.89±5.68	HC	89.97±9.45	HC	89.09±10.29
	MS	81.26±7.71	MS	76.73±13.84	MS	89.46±4.10	MS	70.05±16.60
	p = 0.011		p = 0.0		p = 0.779		p = 0.004	
	**GCL+ LAYER**							
	G1		G2		G3		G4	
	HC	45.31±3.92	HC	44.55±3.10	HC	44.10±3.86	HC	42.93±3.50
	MS	42.93±3.17	MS	42.10±4.57	MS	39.93±3.15	MS	36.94±7.37
	p = 0.095		p = 0.01		p = 0.002		p = 0.018	

Healthy persons and MS patients were distributed in four groups attending to their age. These groups were classified in the following form: G1: 20–34 years old; G2: 35–49 years old; G3: 50–64 years old and G4: 65–80 years old. Fig 2 shows the evolution of the average thickness for different age patients both for HCs and MS patients. It can be seen that the thickness for both retinal layers was always smaller for MS patients than HCs for all ages. Moreover, while for HCs the same trend can be observed for both layers among age groups, MS patients showed an irregular behavior. The three analyzed protocols obtained similar results, a thinning of RNFL and GCL+ of MS patients for all age groups compared to the HCs.

Table 1 shows the results of the t-Student statistical analysis. P-values lesser than p = 0.05 are highlighted. It can be observed that more significant differences are obtained between HCs and MS patients for macular and peripapilar protocols than for wide protocol. Furthermore, both layers performed similarly. However, a more complex analysis should be performed to establish which measurement protocol and which layer would be the best to help in MS diagnosis.

### Machine learning algorithms

Different machine learning algorithms were analyzed both for RNFL and GCL+. In this section, only the most relevant results are commented. For an extended information of the performance of other algorithms, refer to the Supplementary Material (S1 Table).

**Fig 3:**
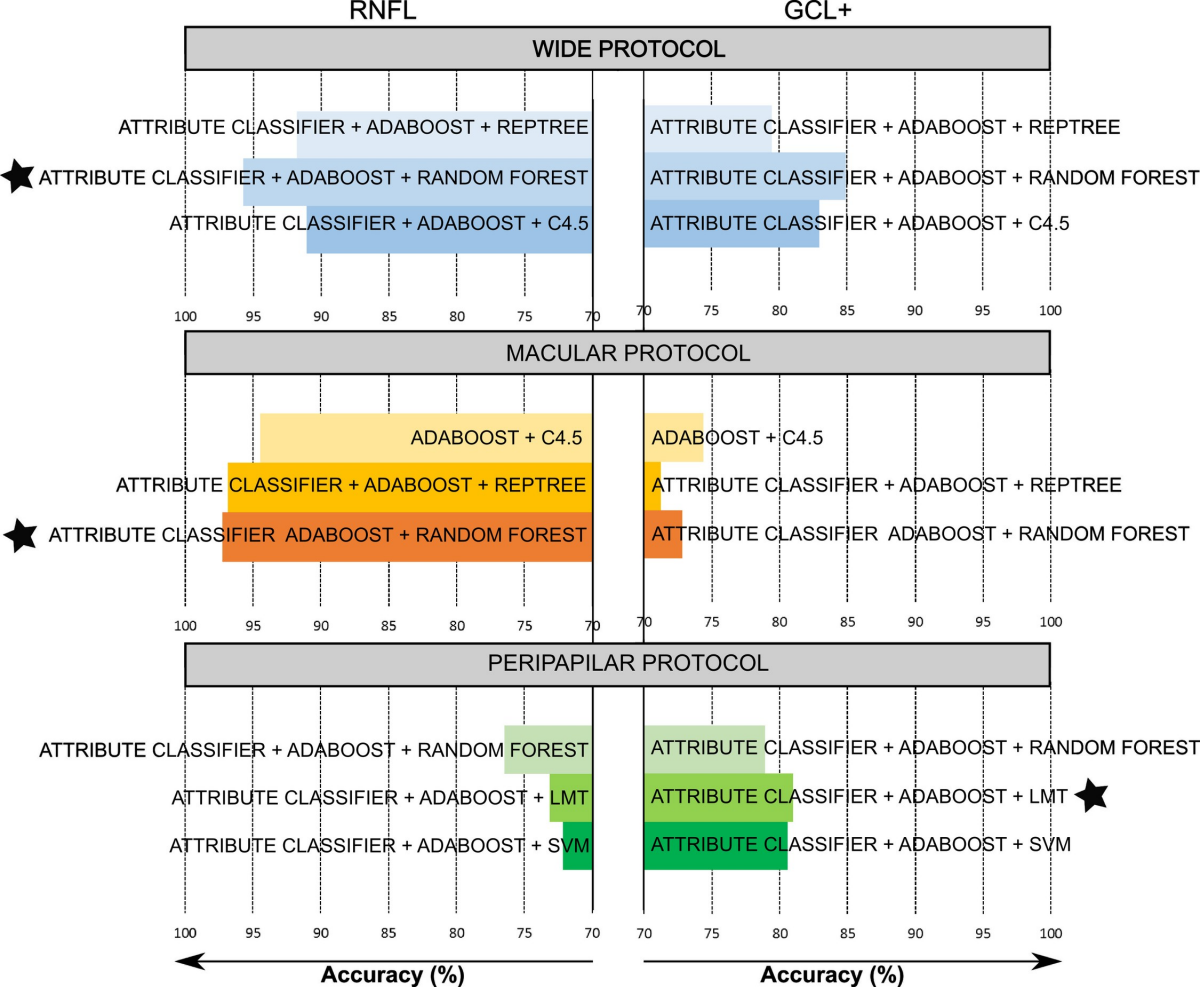
Accuracy of different machine learning algorithms to classify between healthy controls and multiple sclerosis patients. The accuracy of the different machine learning algorithms is shown for the different protocols and for each layer. On the left, results are presented for retinal nerve fiber layer (RNFL) and on the right for the complex ganglion cell layer–inner plexiform layer (GCL+). Only the best algorithms are shown for each analyzed protocol (wide protocol, macular protocol and peripapilar protocol). The results for different decision trees algorithms (RepTree: fast decision tree learner, Random Forest, C4.5 and LMT: logistic model tree) and support vector machine (SVM) are plotted. A star has been placed to highlight the best machine learning algorithm for each protocol.

A schematic representation of the best performance of different algorithms for both layers is shown in Fig 3. In general, it can be seen that the best performance is obtained for the macular protocol using data of the RNFL. Wide protocol which covers a higher area between macula and papilla gives also a good prediction using RNFL data. However, the peripapilar area of the RNFL does not give valuable data for the machine learning algorithms to predict the disease. Although the accuracy is slightly better when using GCL+ data in this area, the value is not relevant (81%).

Analyzing in detail each protocol, the macular protocol (30x30 cube grid centered in the macula) using RNFL data resulted to be the best for predicting MS. Thus, the decision trees combined with an attribute classifier and Adaboost algorithm gave accuracies higher than 95%. In particular, the random forest obtained 97.24% precision. Conversely, ganglion cell layer would not be suitable to predict this disease in this area, since predictions were lower than 75%. Other machine learning algorithms (see supplementary material), as for example, support vector machines (SVM) give 91% accuracy. However, the accuracy of the decision trees was higher in all cases. Notwithstanding the fact that the random forest combined with other algorithms cannot be represented, a C4.5 decision tree has been included in the Supplementary material (S2 Fig) to show the classification process in a tree diagram.

With respect to the wide protocol, the same trend was obtained. Ganglion cell layer does not give enough information to construct powerful machine learning algorithms. However, decision trees present high accuracy to predict the disease using RNFL. The results are shown in Fig 3, and again the combination of attribute classifier, Adaboost algorithm and different decision trees gave accuracies higher than 92%. In detail, random forest had an accuracy of 93.38%.

Finally, as mentioned before, the peripapilar protocol resulted very weak in order to predict MS disease. Curiously, in this protocol obtained accuracies are better for GCL+ than RNFL, however they are still too low.

**Fig 4:**
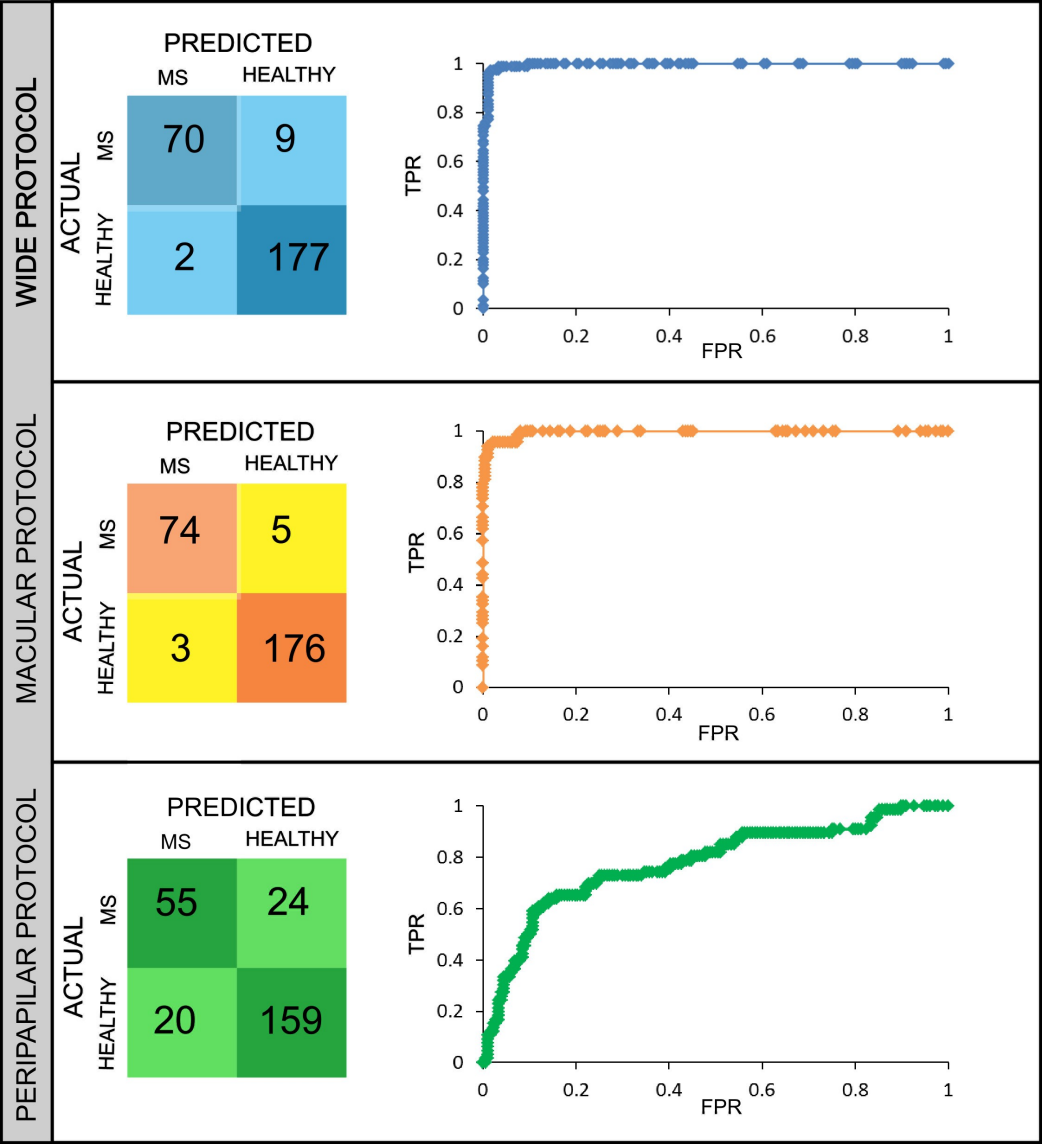
Confusion matrix and ROC curve of the best algorithm for each protocol. On the left, confusion matrix is shown for wide, macular and peripapilar protocol. On the right, receiver-operating characteristic (ROC) curve [on the x-axis the false positive rate (FPR) and on the y-axis the true positive rate (TPR) are represented] for each protocol. In detail, for this representation, the random forest algorithm results have been selected for wide and macular protocol using retinal nerve fiber layer (RNFL) data. For peripapilar protocol, the logistic model tree (LMT) with GCL+ data has been chosen.

The confusion matrix was represented (Fig 4) for the best algorithm for each protocol (these are highlighted in Fig 3) in order to see the performance of the classification model. In our developments, there are two possible predicted classes: MS patient or HC. The confusion matrix represents the true positives (TP) and the false positives (FP), which means in how many cases the algorithm correctly or incorrectly classifies the MS patients. In the second row, the false negatives (FN) and true negatives (TN) are represented, which means how many times the class healthy is incorrectly/correctly classified. It can be seen that the ratio between FP and TP was lowest using the macular protocol for RNFL data.

Regarding to the receiving operating curve (ROC), again the best algorithms were selected and the ROC curve was plotted for each protocol (Fig 4). The ROC curve is created by plotting the true positive rate (TPR) against the false positive rate (FPR) at various thresholds settings. The ROC area was very high for wide (0.998) and for macular (0.995) protocols as can be seen on the right part of Fig 4. However, the ROC area was very low (0.775) for the peripapilar protocol.

On Table 2, different precision parameters are listed for the best algorithms highlighted on Fig 3. It can be seen that the best precision (when the algorithm predicts the disease, how often is correct) is achieved for the macular protocol and also the error rate is the lowest.

**Table 2:** Different precision parameters are listed for each protocol. Only the best algorithms (highlighted in Fig 3) are shown. Accuracy is calculated as (TP+TN)/total; Sensitivity is TP/actual MS; Specificity as TN/actual Healthy; Precision as TP/predicted MS; Error rate as 1 –accuracy and Prevalence as Actual MS/total with total = TN + TP + FP + FN. Abbreviations: TP, true positives; TN, true negatives; FP, false positives; FN, false negatives; MS, multiple sclerosis.

PROTOCOL	ACCURACY	SENSITIVITY	SPECIFITY	PRECISION	ERROR RATE	PREVALENCE
**Wide**	95.74%	97.22%	95.16%	88.61%	4.26%	27.91%
**Macular**	97.24%	95.52%	97.86%	94.12%	2.76%	26.38%
**Peripapilar**	80.99%	67.24%	85.33%	59.09%	19.01%	23.97%

**Fig 5:**
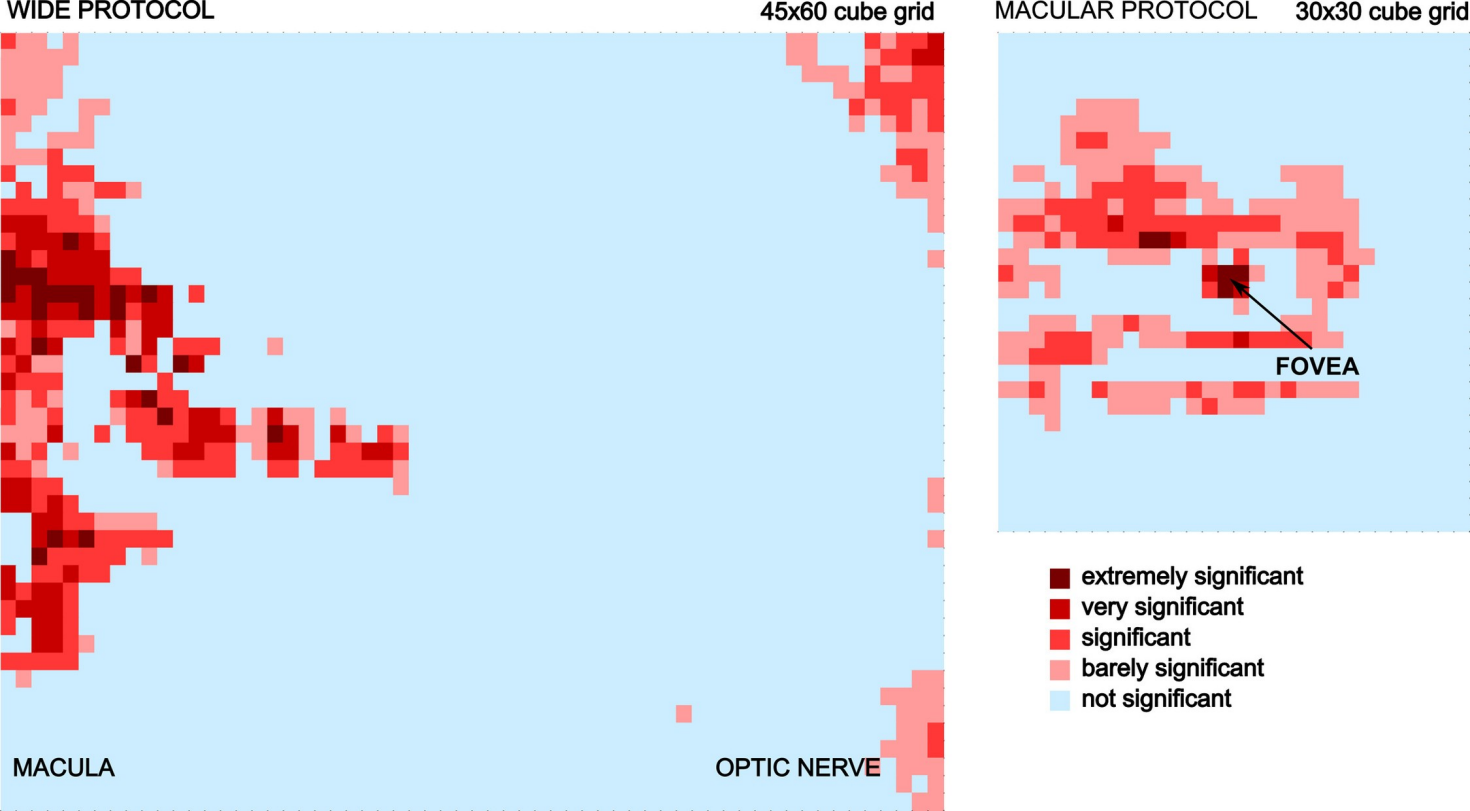
Significance of each cube of the grid for wide and macular protocols for retinal nerve fiber layer (RNFL) to predict multiple sclerosis (MS) disease. On the left the grid of the wide protocol is shown and the most significant areas to predict the disease located at the macula are plotted. On the right, the grid of the macular protocol is shown, and the fovea is highlighted as the most determinant area to detect the disease.

Finally, and attribute weighting algorithm was performed in order to stablish which areas of the retina are more significant to predict MS disease. Since only wide and macula protocols gave relevant results, this analysis has been performed only for these two protocols. The significance of each cube of the grid is reflected in Fig 5. It can be seen that the most significant part of the retina to detect the disease coincide with the macula in both protocols.

## Discussion

Machine learning algorithms have a long history of development and successful application in many scientific fields, improving the ability of clinicians to predict patient outcome in different pathologies [25][26][27][28]. The goal of this work was to analyze the ability of these algorithms to classify between healthy controls and multiple sclerosis patients using SS-OCT data. For that, DRI OCT Triton was used and different protocols were studied to measure RNFL and GCL thicknesses.

Before data mining analysis, RNFL and GCL+ data were statistically treated in order to see differences between HCs and MS patients and among different age groups. It was observed that both layers were thinner in MS patients and this result was obtained for all age groups. These results are in accordance with previous investigations using spectral domain OCT [8][29][26][30]. However, differences between age groups have not been previously described. In our study, more significant differences were obtained between patients and controls using data from macular and peripapilar protocols than from wide protocol. For this comparison, average thickness and standard deviation was used and this simplification does not seem to be suitable when the analyzed region is large, as happens for wide protocol. Attending to age groups, significant differences in GCL+ thickness between groups were obtained for patients older than 35 years old, while for RNFL these differences were more significant for younger people (20–50 years old). Although this work is a cross-sectional study, these results seem to be in accordance with Petzold et al. [8] who observed that the highest annual atrophy rate was found in MS patients with shorter disease duration. Thus, these patients (groups 1 and 2) would have more intact neuroaxonal tissue to lose than patients with longer disease duration [31], and therefore the thickness reduction in patients from 20 to 50 years old would be more significant. In the same line, Balk et al. [10] demonstrated in a longitudinal study that injury in the innermost retinal layers is found in MS and that this damage occurs most rapidly during the early stages of disease. However, it seems clear that the differences between patients and controls are present in all age groups, and both RNFL and GCL+ thicknesses would give valuable information to predict MS disease.

To deeply investigate on these differences, different data mining algorithms were used. This analysis was made using three different protocols provided by DRI OCT Triton. DRI OCT Triton presents one advantage respect to other OCT devices. The information of retinal layers thickness is very precise since the values of the layer thickness is computed in each box of the grid. Thus, the information of each box can be used for the analysis and no average value has to be computed. The highest measure accuracy is obtained for denser grids with smallest box size (200μmx200μm). Therefore, the selected protocols were those centered in the macula (macular protocol), in the papilla (peripapilar protocol) and an extended one covering macular and papilla area (wide protocol). Only measures of RNFL and GCL+ were treated.

Different machine learning algorithms were used for each protocol, and different accuracies were obtained. However, the same two conclusions can be obtained. The first one, is that RNFL data provide more information to classify between MS and healthy patients than GCL+ data. The second conclusion is that both macular and wide protocols are more useful for data mining analysis than peripapilar protocol.

In general, the best accuracy was obtained for the decision tree algorithms. As it can be seen in the supplementary material a wide range of algorithms have been tested for every protocol. For instance, Multilayer Perceptron does not give high accuracy (82.2% for macular protocol using RNFL data). On the other hand, support vector machines (SVM) are supposed to be the best binary classifiers because theoretically SVM get the best possible solution and avoid local minima related solutions. However, in our calculations, the highest SVM accuracy was 90.94% (for macular protocol using RNFL data) and ROC area of 0.931. These values are very far from decision tree results: 95.73% accuracy and 0.998 ROC area for wide protocol and 97.24% accuracy and 0.995 ROC area for macular protocol. These results were obtained using the random forest algorithm combined with Adaboost algorithm for both protocols. Although, this type of algorithm cannot be easily represented, S2 Fig shows a C4.5 decision tree for macular protocol using RNFL data. It can be appreciated which boxes of the grid are determinant to predict the disease, and it was obtained that only 8 boxes were used to obtain an accuracy of the classification algorithm of 92.92% and ROC Area of 0.934.

Attending to the best precision obtained, this was obtained for macular protocol using RNFL data, and with the combination of an attribute classifier, Adaboost algorithm and random forest algorithm. The use of Adaboost algorithm improves significantly the accuracy of the algorithms, in special of the decision trees.

As previously mentioned, macular area of the retinal nerve fiber layer provides relevant data to construct a classification between healthy and MS patients. Therefore, an analysis of which specific areas are more significant to predict the disease was performed. Fig 5 shows a color map indicating the most significant areas both for macular and wide protocols. It can be seen how the macular zone is determinant to detect MS disease. Furthermore, the significance of each cube of the grid can be analyzed, and the temporal and superior zones of the macula were very significant to predict the disease. These locations correspond to the location of small RGCs which are more susceptible to be damaged than larger ones [32]. Our results are totally in accordance with other works [33][34].

On the other hand, GCL+ did not contribute to predict MS and the different machine learning techniques performed worse in this layer than in RNFL. Although this result may result surprising since GCL+ thickness in MS patients has been shown to correlate better than RNFL thickness with EDSS score [12][31], it has been previously reported [29][35][36]. Notwithstanding the fact, that the thickness of both layers (RNFL and GCL+) decreases for MS patients (see Fig 2 and Table 1), in this work, the data mining process obtained more reliable rates when using RNFL data. Considering that, the purpose of this research is to construct a classifier to distinguish between MS patient or HC, the EDSS score was not considered as an input of the algorithm. Therefore, the possible relation between disease disability and GCL+ or RNFL thickness could not be taken into account.

In this work, an exhaustive evaluation of different machine learning algorithms was performed. This kind of research has only be made before using MRI data to predict MS course [37][38], and in these papers, SVM and k-nearest neighbors (kNN) respectively performed better than other classifiers. Our work is the first which analyses swept-source OCT data to classify between MS patients and healthy controls, and the decision trees performed as powerful classifiers. The quality of the data obtained by the OCT platforms is influenced by retinal pigment epithelium status and media opacity. In addition, swept-source OCT gives real thickness values in each grid box and therefore the number of inputs for the data mining process is higher than in spectral domain OCT measurements. Thus, the use of decision trees could correctly identify a high percentage of MS subjects. In our study, we selected only good-quality scans, but in clinical practice, this is not always possible. These limitations must be taken into account when interpreting OCT and data mining results.

We have found that the combination of some RNFL thickness measurements obtained with swept-source OCT provides high precision to detect the disease using machine learning algorithms. This can be considered as a practical tool to help clinicians for discriminating between normal and MS patients with early diagnosis or nondefinitive MS diagnosis. Here, promising results have been obtained, however, MS cohort has average disease duration of 7.12 years and therefore the classification between healthy controls and MS patients is easier than using only data from the onset of the disease. Thus, MS cohort should be modified in the future only considering patients with at least only one year of disease duration.

In spite of the relevant information that can be extracted from OCT measures, nowadays the diagnosis of MS is determined by a neurologist based on standard clinical and neuroimaging criteria (2017 Mc Donald criteria) [15]. Even though MRI is a reliable tool to diagnose MS, the definite diagnosis can take years since not every patient’s symptoms and signs fit with McDonald criteria’s parameters. Thus, without a single ‘gold standard’ diagnostic test, MS is a difficult disease to describe precisely. Moreover, there are new disease-modifying treatments (alemtuzumab, cladribina or ocrelizumab) that would help to stop MS progression and neural damage if an early diagnosis could be made. Several authors have proposed that MS diagnostic criteria should include OCT parameters [7] [9][39]. Our work confirms that the use of OCT parameters (specifically RNFL thickness) can predict the disease with a precision higher than 95%.

In conclusion, a review of MS diagnostic criteria, including RNFL measurements provided by OCT and machine learning analysis, may improve the sensitivity–specificity balance in diagnostic performance.

## Supporting information

S1 Table The accuracy and the receiver-operating characteristic (ROC) area obtained for different machine learning algorithms for wide, macular and peripapilar protocols. The following notation has been used: **AC**: Attribute classifier; **ADA**: Adaboost; **C4.5**: Decision Tree; **BAG**: Bagging; **DS**: Decision Stump; **HT**: Hoeffding Tree; **LMT**: Logistic model tree; **MLP**: Multilayer Perceptron; **RepTree**: Fast Decision Tree Learner; **RF**: Random Forest; **RT**: Random Tree; **SVM (C-SVC)**: Support Vector Machine with c value ranging from 0 to infinity; **SVM (NU-SVC)**: Support Vector Machine with nu value ranging from 0 to 1.
(DOCX)

S1 Fig Average RNFL and GCL+ thickness for healthy controls and MS patients. Average thickness of retina nerve fiber layer (RNFL) and the complex ganglion cell layer–inner plexiform layer (GCL+) for wide, macular and peripapilar protocols. On the left, data for the healthy patients are shown; on the right, data correspond to multiple sclerosis (MS) patients. The median and the quartiles are shown.
(TIF)

S2 Fig Scheme of C4.5 decision tree using RNFL data to classify between healthy controls (HC) and multiple sclerosis (MS) patients. C4.5 decision tree for macular protocol (30x30 cube grid centered on the macula) using retinal nerve fiber layer (RNFL) data. The accuracy of the classification algorithm is 92.92% and receiver-operating characteristic (ROC) Area 0.934. It can be seen that the first test corresponds to the component 14_15 of the grid (shown on the right part of the picture), and the algorithm is able to predict 29 multiple sclerosis (MS) patients. The next level is constructed based on the value of the 10_13 box. Depending on this vale, two new branches appear. Continuing like this, the decision tree classifies healthy patients (H) and MS patients. The value of each attribute is normalized. It can be seen that the most significant box is 14_15 which corresponds to fovea location.
(TIF)
