# Supplementary material for: Swept source optical coherence tomography to early detect multiple sclerosis disease. The use of machine learning techniques
Source: PLoS One. 2019 May 6;14(5):e0216410. doi: 10.1371/journal.pone.0216410 (PMC6502323; doi:10.1371/journal.pone.0216410)
Supplement: S1 Table — The following notation has been used: AC: Attribute classifier; ADA: Adaboost; C4.5: Decision Tree; BAG: Bagging; DS: Decision Stump; HT: Hoeffding Tree; LMT: Logistic model tree; MLP: Multilayer Perceptron; RepTree: Fast Decision Tree Learner; RF: Random Forest; RT: Random Tree; SVM (C-SVC): Support Vector Machine with c value ranging from 0 to infinity; SVM (NU-SVC): Support Vector Machine with nu value ranging from 0 to 1. (DOCX) [file pone.0216410.s001.docx]

| **WIDE PROTOCOL** | | | | | | | | | | |
| --- | --- | --- | --- | --- | --- | --- | --- | --- | --- | --- |
| **RNFL** |  |  |  |  |  | **GLC+** |  |  |  |  |
| **ALGORITHM** |  |  | **%** | **ROC AREA** |  | **ALGORITHM** |  |  | **%** | **ROC AREA** |
| ADA+C4.5 |  |  | 89.14 | 0.95 |  | ADA C4.5 |  |  | 75.58 | 0.751 |
| AC+ADA+C4.5 |  |  | **91.08** | **0.979** |  | AC+ADA+C4.5 |  |  | **82.97** | **0.854** |
| ADA+SVM (C-SVC) | |  | 68.21 | 0.548 |  | ADA+SVM (C-SVC) | |  | 63.95 | 0.502 |
| ADA+SVM (NU-SVC, NU=0.5) | | | 67.82 | 0.606 |  | ADA+SVM (NU-SVC, NU=0.5) | | | 62.01 | 0.535 |
| ADA+SVM (NU-SVC, NU=0.25) | | | 69.76 | 0.586 |  | ADA+SVM (NU-SVC, NU=0.25) | | | 62.79 | 0.506 |
| AC+ADA+SVM (C-SVC) | | | 83.72 | 0.882 |  | AC+ADA+SVM (C-SVC) | |  | 71.31 | 0.728 |
| AC+ADA+SVM (NU-SVC, NU=0.25) | | | 81.78 | 0.864 |  | AC+ADA+SVM (NU-SVC, NU=0.25) | | | 71.31 | 0.728 |
| AC+SVM (C-SVC) |  |  | 77.51 | 0.64 |  | AC+SVM (C-SVC) | |  | 71.31 | 0.567 |
| BAG+SVM (C-SCV) | |  | 70.5 | 0.559 |  | BAG+SVM (C-SCV) | |  | 70.54 | 0.558 |
| AC+BAG+SVM (C-SVC) | | | 76.74 | 0.722 |  | AC+BAG+SVM (C-SVC) | |  | 73.34 | 0.683 |
| AC+ADA+MLP |  |  | 70.15 | 0.674 |  | AC+ADA+MLP |  |  | 63.56 | 0.645 |
| AC+ADA+LG |  |  | 63.95 | 0.221 |  | AC+ADA+LG |  |  | 65.116 | 0.186 |
| AC+ADA+DS |  |  | 86.04 | 0.927 |  | AC+ADA+DS |  |  | 75.19 | 0.768 |
| AC+ADA+HT |  |  | 68.6 | 0.566 |  | AC+ADA+HT |  |  | 69.76 | 0.539 |
| AC+ADA+LMT |  |  | 92.24 | 0.979 |  | AC+ADA+LMT |  |  | 77.51 | 0.792 |
| AC+ADA+RF |  |  | **95.73** | **0.998** |  | AC+ADA+RF |  |  | **84.88** | **0.882** |
| AC+ADA+RT |  |  | 86.82 | 0.835 |  | AC+ADA+RT |  |  | 76.35 | 0.709 |
| AC+ADA+REPTREE | |  | **91.8** | **0.971** |  | AC+ADA+REPTREE | |  | **79.45** | **0.8** |
|  |  |  |  |  |  |  |  |  |  |  |
| **MACULAR PROTOCOL** | | | | | | | | | | |
| **RNFL** |  |  |  |  |  | **GLC+** |  |  |  |  |
| **ALGORITHM** |  |  | **%** | **ROC AREA** |  | **ALGORITHM** |  |  | **%** | **ROC AREA** |
| ADA+C4.5 |  |  | **94.48** | **0.964** |  | ADA+C4.5 |  |  | **74.4** | **0.681** |
| AC+ADA+C4.5 |  |  | 95.66 | 0.987 |  | AC+ADA+C4.5 |  |  | 46.06 | 0.138 |
| ADA+SVM (C-SVC) | |  | 80.31 | 792 |  | ADA+SVM (C-SVC) | |  | 71.25 | 0.593 |
| ADA+SVM (NU-SVC, NU=0.5) | | | - | |  | ADA+SVM (NU-SVC, NU=0.5) | | | - | |
| ADA+SVM (NU-SVC, NU=0.25) | | | 83.027 | 0.809 |  | ADA+SVM (NU-SVC, NU=0.25) | | | 71.25 | 0.576 |
| AC+ADA+SVM (C-SVC) | | | 88.18 | 0.906 |  | AC+ADA+SVM (C-SVC) | |  | 68,11 | 0.599 |
| AC+ADA+SVM (NU-SVC, NU=0.25) | | | 90.94 | 0.931 |  | AC+ADA+SVM (NU-SVC, NU=0.25) | | | 68.11 | 0.632 |
| AC+SVM (C-SVC) |  |  | 86.61 | 0.773 |  | AC+SVM (C-SVC) | |  | 72.44 | 0.495 |
| BAG+SVM (C-SCV) | |  | 77.55 | 0.637 |  | BAG+SVM (C-SCV) | |  | 73.22 | 0.555 |
| AC+BAG+SVM (C-SVC) | | | 85.43 | 0.86 |  | AC+BAG+SVM (C-SVC) | |  | 72.83 | 0.539 |
| AC+ADA+MLP |  |  | 82.28 | 0,789 |  | AC+ADA+MLP |  |  | 72.83 | 0.588 |
| AC+ADA+LG |  |  | 70.47 | 0.277 |  | AC+ADA+LG |  |  | 73.22 | 0.477 |
| AC+ADA+DS |  |  | 96.07 | 0.992 |  | AC+ADA+DS |  |  | 72.44 | 0.616 |
| AC+ADA+HT |  |  | 72.04 | 0.556 |  | AC+ADA+HT |  |  | 73.22 | 0.473 |
| AC+ADA+LMT |  |  | 96.45 | 0.987 |  | AC+ADA+LMT |  |  | 72.44 | 0.64 |
| AC+ADA+RF |  |  | **97.24** | **0.995** |  | AC+ADA+RF |  |  | 72.83 | 0.676 |
| AC+ADA+RT |  |  | 92-51 | 0.897 |  | AC+ADA+RT |  |  | 68.11 | 0.61 |
| AC+ADA+REPTREE | |  | **96.85** | **0.978** |  | AC+ADA+REPTREE | |  | **71.25** | **0.679** |
|  |  |  |  |  |  | ADA+RF |  |  | **74.01** | **0.71** |
|  |  |  |  |  |  |  |  |  |  |  |
| **PERIPAPILAR PROTOCOL** | | | | | | | | | | |
| **RNFL** |  |  |  |  |  | **GLC+** |  |  |  |  |
| **ALGORITHM** |  |  | **%** | **ROC AREA** |  | **ALGORITHM** |  |  | **%** | **ROC AREA** |
| ADA+C4.5 |  |  | 72.31 | 0.635 |  | ADA+C4.5 |  |  | 73.14 | 0.72 |
| AC+ADA+C4.5 |  |  | 74.79 | 0.726 |  | AC+ADA+C4.5 |  |  | 76.03 | 0.726 |
| ADA+SVM (C-SVC) | |  | 69 | 0.585 |  | ADA+SVM (C-SVC) | |  | 66.11 | 0.554 |
| ADA+SVM (NU-SVC, NU=0.5) | | | 69.42 | 0.586 |  | ADA+SVM (NU-SVC, NU=0.5) | | | 63,63 | 0.582 |
| ADA+SVM (NU-SVC, NU=0.25) | | | 69 | 0.581 |  | ADA+SVM (NU-SVC, NU=0.25) | | | 65.28 | 0.563 |
| AC+ADA+SVM (C-SVC) | | | 72.13 | 0.644 |  | AC+ADA+SVM (C-SVC) | |  | **80.57** | **0.808** |
| AC+ADA+SVM (NU-SVC ,NU=0.25) | | | **73.55** | **0.669** |  | AC+ADA+SVM (NU-SVC, NU=0.25) | | | 78.09 | 0.805 |
| AC+SVM (C-SVC) |  |  | 71.9 | 0.499 |  | AC+SVM (C-SVC) | |  | 77.27 | 0.73 |
| BAG+SVM (C-SCV) | |  | 72.72 | 0.576 |  | BAG+SVM (C-SCV) | |  | 72.31 | 0.55 |
| AC+BAG+SVM (C-SVC) | | | 73.96 | 0.566 |  | AC+BAG+SVM (C-SVC) | |  | 78.09 | 0.822 |
| AC+ADA+MLP |  |  | 71.48 | 0.49 |  | AC+ADA+MLP |  |  | 73.55 | 0.695 |
| AC+ADA+LG |  |  | 71.9 | 0.468 |  | AC+ADA+LG |  |  | 72.31 | 0.416 |
| AC+ADA+DS |  |  | 73.96 | 0.626 |  | AC+ADA+DS |  |  | 70.24 | 0.714 |
| AC+ADA+HT |  |  | 73.14 | 0.562 |  | AC+ADA+HT |  |  | 69.83 | 0.661 |
| AC+ADA+LMT |  |  | **73.14** | **0.708** |  | AC+ADA+LMT |  |  | **80.99** | **0.775** |
| AC+ADA+RF |  |  | **76.44** | **0.78** |  | AC+ADA+RF |  |  | **78.92** | **0.801** |
| AC+ADA+RT |  |  | 72.72 | 0.67 |  | AC+ADA+RT |  |  | 76.44 | 0.678 |
| AC+ADA+REPTREE | |  | 76.01 | 0.774 |  | AC+ADA+REPTREE | |  | 76.03 | 0.762 |

**S1 Table. The accuracy and the receiver-operating characteristic (ROC) area obtained for different machine learning algorithms for wide, macular and peripapilar protocols.**

The following notation has been used: **AC**: Attribute classifier; **ADA**: Adaboost; **C4.5**: Decision Tree; **BAG**: Bagging; **DS**: Decision Stump; **HT**: Hoeffding Tree; **LMT**: Logistic model tree; **MLP**: Multilayer Perceptron; **RepTree**: Fast Decision Tree Learner; **RF**: Random Forest; **RT**: Random Tree; **SVM (C-SVC)**: Support Vector Machine with c value ranging from 0 to infinity; **SVM (NU-SVC)**: Support Vector Machine with nu value ranging from 0 to 1.
