# Supplementary figures and images for: Swept source optical coherence tomography to early detect multiple sclerosis disease. The use of machine learning techniques
Source: PLoS One. 2019 May 6;14(5):e0216410. doi: 10.1371/journal.pone.0216410 (PMC6502323; doi:10.1371/journal.pone.0216410)

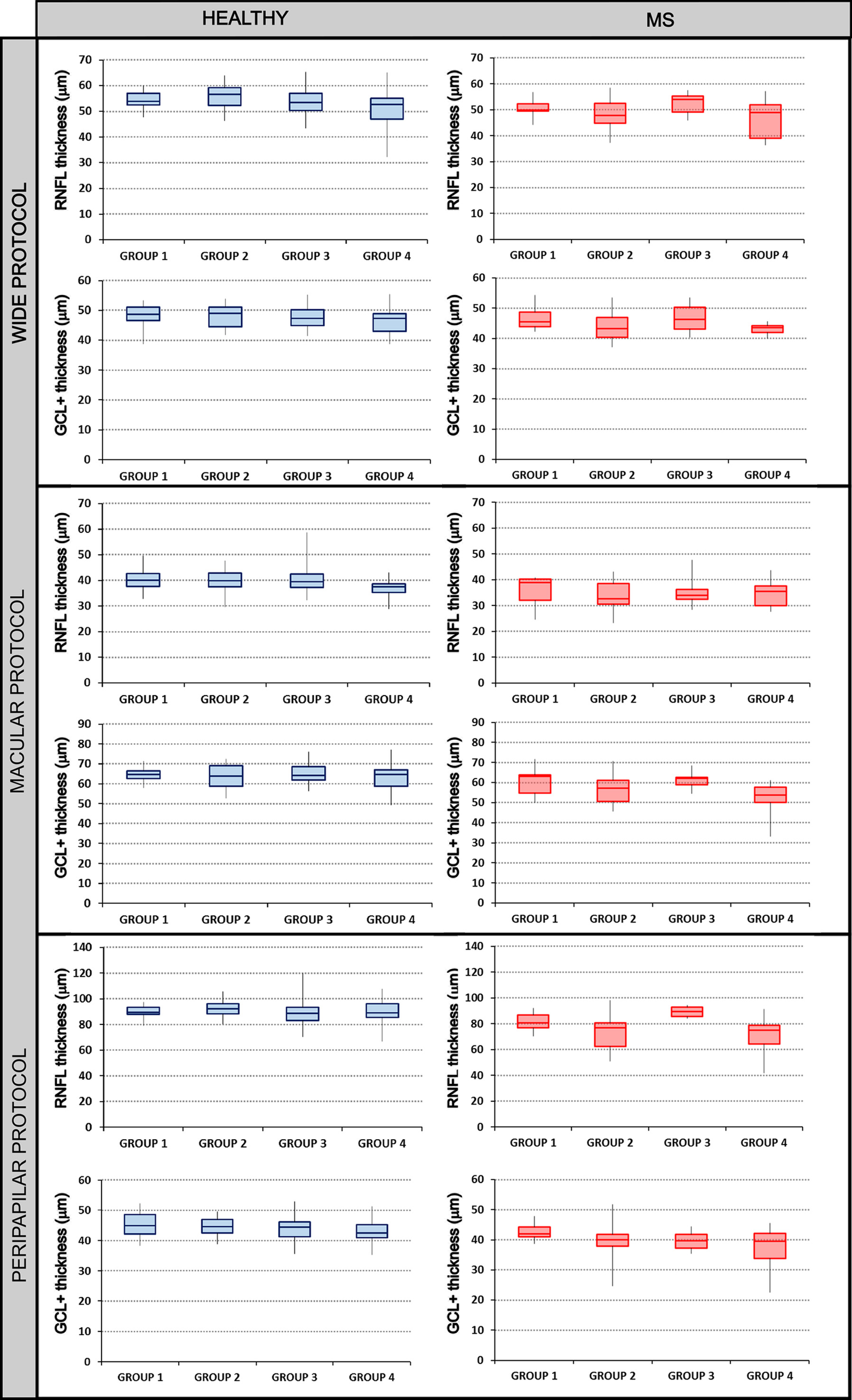

Supplement: S1 Fig — Average thickness of retina nerve fiber layer (RNFL) and the complex ganglion cell layer–inner plexiform layer (GCL+) for wide, macular and peripapilar protocols. On the left, data for the healthy patients are shown; on the right, data correspond to multiple sclerosis (MS) patients. The median and the quartiles are shown. (TIF) [file pone.0216410.s002.tif]

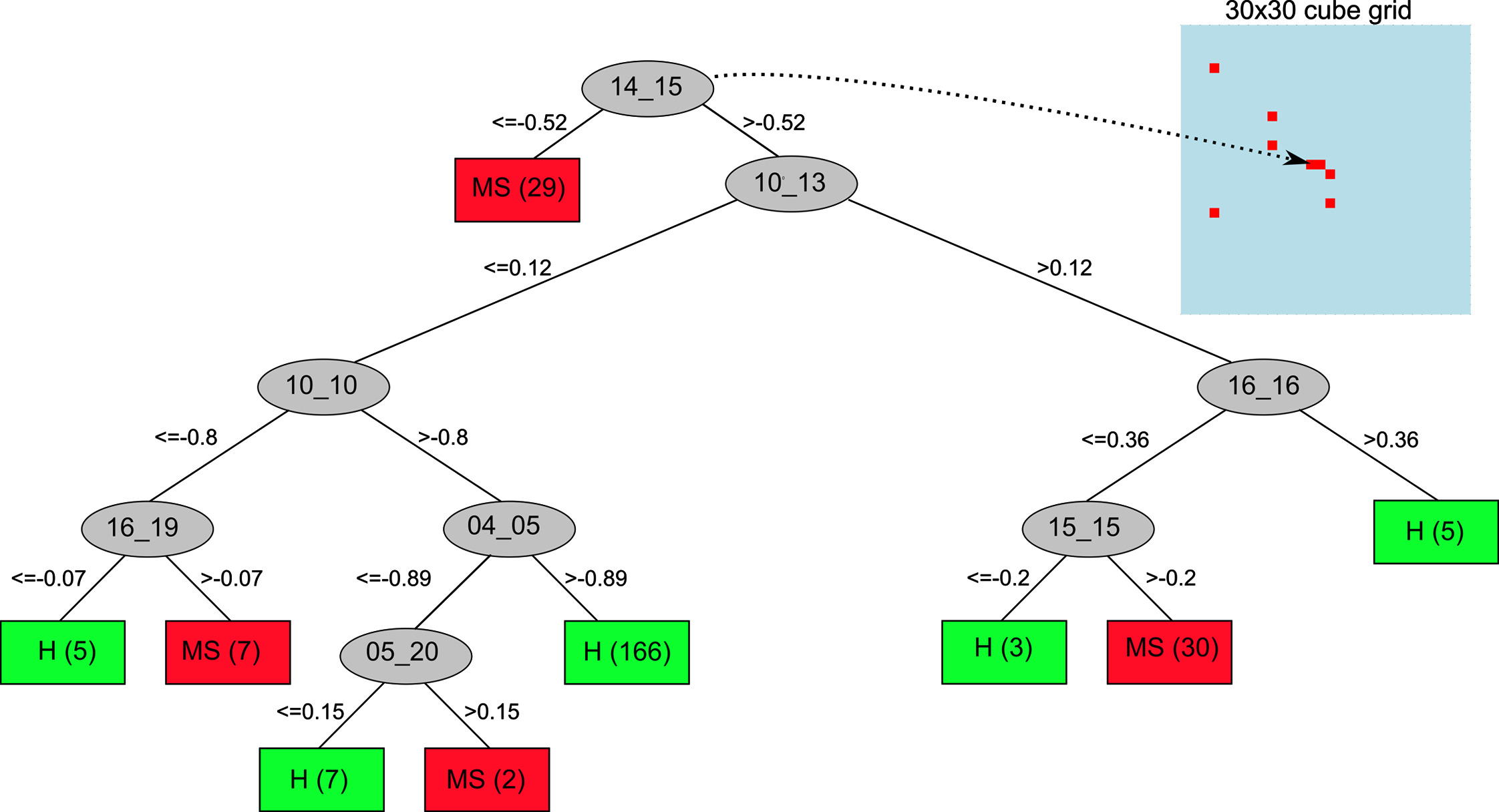

Supplement: S2 Fig — C4.5 decision tree for macular protocol (30x30 cube grid centered on the macula) using retinal nerve fiber layer (RNFL) data. The accuracy of the classification algorithm is 92.92% and receiver-operating characteristic (ROC) Area 0.934. It can be seen that the first test corresponds to the component 14_15 of the grid (shown on the right part of the picture), and the algorithm is able to predict 29 multiple sclerosis (MS) patients. The next level is constructed based on the value of the 10_13 box. Depending on this vale, two new branches appear. Continuing like this, the decision tree classifies healthy patients (H) and MS patients. The value of each attribute is normalized. It can be seen that the most significant box is 14_15 which corresponds to fovea location. (TIF) [file pone.0216410.s003.tif]
